# Cerebro-spinal fluid biomarker levels: phosphorylated tau (T) and total tau (N) as markers for rate of progression in Alzheimer’s disease

**DOI:** 10.1186/s12883-019-1591-0

**Published:** 2020-01-09

**Authors:** Carina Wattmo, Kaj Blennow, Oskar Hansson

**Affiliations:** 1 Clinical Memory Research Unit, Department of Clinical Sciences, Malmö, Lund University, SE-205 02 Malmö, Sweden; 2 Memory Clinic, Skåne University Hospital, SE-205 02 Malmö, Sweden; 3 Institute of Neuroscience and Physiology, Department of Psychiatry and Neurochemistry, the Sahlgrenska Academy, University of Gothenburg, SE-431 80 Mölndal, Sweden

**Keywords:** Alzheimer’s disease, Cholinesterase inhibitors, Cognition, Activities of daily living, Apolipoprotein E, Treatment effect, Disease progression, CSF biomarkers, AT(N), Tau

## Abstract

**Background:**

We investigated the potential associations between cerebro-spinal fluid (CSF) levels of phosphorylated tau (P-tau) and total tau (T-tau) with short-term response to cholinesterase inhibitor (ChEI) treatment, longitudinal outcome and progression rates in Alzheimer’s disease (AD).

**Methods:**

This prospective, observational study included 129 participants clinically diagnosed with mild-to-moderate AD, who underwent a lumbar puncture. The CSF biomarkers amyloid-β_1–42_ (Aβ_42_), P-tau and T-tau were analysed with xMAP technology. Cognitive, global, instrumental and basic activities of daily living (ADL) capacities at the start of ChEI therapy and semi-annually over 3 years were evaluated.

**Results:**

All patients had abnormal Aβ_42_ (A+). Fifty-eight individuals (45%) exhibited normal P-tau and T-tau (A+ T– (N)–), 12 (9%) abnormal P-tau/normal T-tau (A+ T+ (N)–), 17 (13%) normal P-tau/abnormal T-tau (A+ T– (N)+) and 42 (33%) abnormal P-tau and T-tau (A+ T+ (N)+). The participants with A+ T+ (N)+ were younger than A+ T– (N)+ at the estimated onset of AD and the initiation of ChEIs. The proportion of 6-month responders to ChEI and deterioration/year after start of treatment did not differ between the AT(N) profiles in any scales. A higher percentage of globally improved/unchanged patients was exhibited in the A+ T– (N)– group after 12, 30 and 36 months of ChEI therapy but not at other assessments. In apolipoprotein E (*APOE*) ε4-carriers, linear relationships were found between greater cognitive decline/year and higher tau; Mini-Mental State Examination score – T-tau (*r*_*s*_ = − 0.257, *p* = 0.014) and Alzheimer’s Disease Assessment Scale–cognitive subscale – P-tau (*r*_*s*_ = − 0.242, *p* = 0.022). A correlation between faster progression in instrumental ADL (IADL) and higher T-tau was also detected (*r*_*s*_ = − 0.232, *p* = 0.028). These associations were not demonstrated in non-ε4-carriers.

**Conclusions:**

Younger age and faster global deterioration were observed in AD patients with pathologic tau and neurodegeneration, whereas more rapid cognitive and IADL decline were related to higher P-tau or T-tau in *APOE* ε4-carriers only. The results might indicate an association between more pronounced tau pathology/neuronal injury and the *APOE* ε4-allele leading to a worse prognosis. Our findings showed that the AT(N) biomarker profiles have limited utility to predict AD progression rates and, thus, measure change and interpreting outcomes from clinical trials of future therapies.

## Background

The pathological process in Alzheimer’s disease (AD) probably starts decades before the onset of symptoms and the clinical AD diagnosis [1]. AD is characterized by a progressive cognitive decline usually beginning with impairment in episodic memory, and a gradual loss of instrumental, and later, basic activities of daily living (ADL) [2]. According to the amyloid cascade hypothesis, the initiating event in AD pathogenesis is an imbalance between the production and clearance of amyloid-β_1–42_ (Aβ_42_) leading to accumulation of amyloid plaques in the brain that damages the synaptic function and mediates the formation of neurofibrillary tangles [3]. Cerebro-spinal fluid (CSF) total tau (T-tau) has been suggested as a general marker of neurodegeneration [4], while phosphorylated tau (P-tau) may be a more specific marker for AD because neurofibrillary tangles primarily consist of tau protein in the abnormally hyperphosphorylated state [5]. The density of tangles has been reported to correlate better with cognitive deterioration than amyloid plaque load [6].

In AD patients, the level of Aβ_42_ is usually lower, and the levels of T-tau and P-tau are usually higher than in healthy elderly people [7]. However, a large variation in the CSF biomarker levels exists among individuals with AD. Recently, a research framework for biomarker classification was published. The biomarkers were grouped into those of β-amyloid deposition “A”, pathologic tau “T” and neurodegeneration/neuronal injury “(N)” (non-AD specific; thus, labelled in parentheses). With the application of cut points, each of the A, T or (N) can be classified as abnormal (+) or normal (−), resulting in various AT(N) biomarker profiles [8]. A useful biomarker should be related to the person’s cognitive and/or functional capacities to, for example, estimate the clinical onset of AD, predict and monitor the course of the disease over time and analyse the potential response to therapies [9]. It is not known whether these issues differ depending on the AT(N) biomarker profile.

In some longitudinal studies, high T-tau and P-tau have been related to faster cognitive decline [10–12]. Other studies of AD have not detected any associations between CSF biomarkers and cognitive ability [13, 14]. Very few studies have investigated the possible relationships between the biomarkers and instrumental (but not basic) ADL and the findings were inconsistent [15, 16]. Moreover, some previous studies have shown long-term stability of tau in AD, despite changes in the patient’s cognitive and global performance [14, 17], while other studies described that tau levels are increased at follow-up [18, 19]. A recent study using data from the Alzheimer’s Disease Neuroimaging Initiative (ADNI) observed a decreased annual change of P-tau, but not T-tau, in the mild AD cohort [20].

After 20 years, cholinesterase inhibitors (ChEIs) are still the predominant symptomatic treatment for AD. ChEIs prevent the degradation of acetylcholine (ACh) by inhibiting the enzyme acetylcholinesterase, leading to increased levels of ACh in the synaptic cleft available for receptor absorption. This enhances cholinergic transmission and improves the communication between neurons [21]. Our studies and others have noted that a protective factor for better cognitive response to ChEI was lower cognitive status at the start of therapy [22, 23]. Remarkably few AD studies over the years have evaluated the association between various aspects of ChEI treatment and CSF biomarkers. Our group suggested there was a worse cognitive short-term response to therapy and faster deterioration in ChEI-treated individuals with very high levels of T-tau and P-tau [24], which indicate an intense disease with pronounced neurodegeneration. In contrast, the above-mentioned observations of better response to ChEI treatment in more advanced AD could imply greater pathology among the responders. Hence, improved knowledge of the relationships between outcomes of ChEI therapy and levels of CSF biomarkers is essential.

Several phase 3 studies of passive immunization with anti-beta-amyloid antibodies have demonstrated a reduction of brain amyloid in participants with mild-to-moderate AD, but no placebo–treatment differences in cognition or ADL, indicating a lack of correlation between AD pathologies, such as amyloid plaque load and the individual’s disabilities [25, 26]. Clinical trials of immunization with anti-tau antibodies are now ongoing [27].

The current study of AD aims to assess the potential associations between patients with different AT(N) biomarker profiles and: (1) rates of progression and longitudinal prognosis in cognitive, global and functional performance, (2) short-term response to ChEI therapy and (3) the relationship with apolipoprotein E (*APOE*) genotype.

## Methods

### Study and subjects

The present AD cohort of 129 participants was recruited prospectively from the Memory Clinic, Skåne University Hospital, Malmö, Sweden. A subgroup of these patients using the same run and batch of reagents was included in an earlier study which defined CSF biomarker cut-off values [28]. At the initiation of ChEIs (baseline), the individuals in this study underwent a lumbar puncture (LP) and exhibited a Mini-Mental State Examination (MMSE) [29] score ranging from 10 to 26, i.e., mild-to-moderate AD. The cohort is part of the Swedish Alzheimer Treatment Study (SATS); different findings from this study have been presented in several publications, for example, [23, 24, 30–32].

The SATS is a 3-year, open-label, non-randomized, multicentre study performed in a routine clinical setting, which was undertaken to assess the long-term effectiveness of ChEI therapy in everyday outpatients on various aspects of AD (e.g., cognitive, global, functional). Before inclusion, all participants underwent a thorough clinical investigation including medical history, physical and neurological examinations, cognitive evaluations, laboratory tests, and cerebral computed tomography to rule out other causes of dementia. Additionally, in some centres, the individuals were investigated further through measurement of regional cerebral blood flow (Cortexplorer using 133-Xenon inhalation or single-photon emission computed tomography), electroencephalography, and neuropsychological tests. Patients fulfilling the clinical criteria of dementia, as defined by the *Diagnostic and Statistical Manual of Mental Disorders*, 4^th^ edition (DSM-IV) [33], and those of probable or possible AD, according to the National Institute of Neurological and Communicative Disorders and Stroke and the Alzheimer’s Disease and Related Disorders Association (NINCDS-ADRDA) [34] were included in the SATS. All participants were diagnosed and subsequently followed up by clinicians specialized in dementia disorders. Additional inclusion criteria were: older than 40 years, living at home at the time of diagnosis, having a responsible caregiver, and assessable with the MMSE at baseline. Individuals not fulfilling the diagnostic criteria for AD, those already undergoing active treatment with any ChEI drug or patients with contra-indications for ChEI therapy were excluded from the study. The contra-indications for ChEIs are cardiac conduction diseases, such as sick sinus syndrome. Caution is required in people with severe asthma or chronic obstructive pulmonary disease, history of peptic ulcers, and severe liver or kidney disease.

Shortly after diagnosis of AD, the patients were enrolled in the study and performed the baseline evaluations; then they received ChEI treatment according to the approved product labelling, as in routine clinical practice. All decisions regarding drug agent and dose were left to the individual clinicians and all dose adjustments were recorded throughout the study.

### Assessment scales

The SATS patients were assessed in a well-structured, follow-up programme using cognitive, global and functional rating scales at the initiation of ChEI therapy, after 2 months (MMSE and global scores only) and every 6 months over 3 years. Cognitive status was evaluated using the MMSE, with scores ranging from 0 to 30 (a higher score indicating less impaired cognition), and the Alzheimer’s Disease Assessment Scale–cognitive subscale (ADAS-cog) [35], with a total score ranging from 0 to 70 (a higher score indicating more impaired cognition). The Clinician Interview-Based Impression of Change (CIBIC) [36] was used as a global rating of “change from the start of ChEI treatment”. The assessments were based on the dementia specialist’s clinical judgement and were performed at all intervals using a 7-point scale that varied from 1 (very much improved) to 7 (marked worsening). Three groups of response were defined at each CIBIC interval: 1–3 indicated improvement, 4 indicated no change and 5–7 indicated worsening.

The Instrumental Activities of Daily Living (IADL) scale [37] consists of eight different items: ability to use the telephone, shopping, food preparation, housekeeping, laundry, mode of transportation, responsibility for own medications and ability to handle finances. Each item was scored from 1 (no impairment) to 3–5 (severe impairment), which allowed a total range of 8–31 points. The Physical Self-Maintenance Scale (PSMS) [37] consists of six different items: toilet, feeding, dressing, grooming, physical ambulation and bathing. Each item was scored from 1 (no impairment) to 5 (severe impairment), which yielded a total range of 6–30 points. Trained dementia nurses obtained the ADL performance from interviews with each caregiver. Response was calculated as the change in score between the 6-month follow-up after the initiation of ChEI therapy and the baseline for each scale (MMSE, ADAS-cog, IADL or PSMS). The annual rate of progression, i.e., the change in score from baseline to the person’s last evaluation, was divided by the number of months between these visits and multiplied by 12. To facilitate the comparison of rates in MMSE, ADAS-cog, IADL and PSMS scores, changes in score were converted to positive values, which were indicative of improvement, and negative values, which were indicative of decline.

### Analysis of baseline CSF

CSF was collected in polypropylene tubes, stored at − 80 °C and analysed after the clinical follow-up of the study was completed. LP was only performed at the baseline visit. The procedure followed the Alzheimer’s Association Flow Chart for LP and CSF sample processing [38, 39]. The levels of Aβ_42_, P-tau phosphorylated at Thr_181_ and T-tau were determined using xMAP technology [40]. Abnormal levels of CSF biomarkers were defined as Aβ_42_ < 209 ng/ml (A+), P-tau > 51 ng/ml (T+) and T-tau > 100 ng/ml (N)+ [28].

### Statistical analyses

The IBM Statistical Package for the Social Sciences (SPSS) for Windows (version 24.0; IBM Corporation, Armonk, NY, USA) was used to perform the statistical analyses. The level of significance was defined as *p* < 0.05 if not otherwise noted, and all tests were two-tailed. Observed-case analyses were used to avoid overestimation of the treatment effect by imputing better previous outcome scores in a longitudinal study of a progressively advancing disease. One-way analysis of variance (ANOVA) with Bonferroni correction was used to compare the difference between the mean scores calculated from the continuous assessment scales and the four AT(N) biomarker profiles. To compare the quartile or quintile of individuals with the lowest values of Aβ_42_ or the highest values of tau as the reference against all other groups, ANOVA with Dunnett *t* tests was performed. Independent-sample *t* tests were used to compare the differences between the means obtained for two groups, such as *APOE* genotype, and chi-squared tests were computed to analyse categorical variables. Spearman’s non-parametric correlation coefficient was calculated to investigate the presence of any linear associations between the CSF biomarker values and the rates of cognitive and functional deterioration.

## Results

### Baseline characteristics according to AT(N) biomarker profiles

All 129 SATS participants had abnormal (low) CSF Aβ_42_ (A+). The socio-demographic and clinical characteristics of the patients were divided into four biomarker profiles and are displayed in Table 1: normal P-tau and T-tau (A+ T– (N)–), *n* = 58 (45%); abnormal (high) P-tau and normal T-tau (A+ T+ (N)–), *n* = 12 (9%); normal P-tau and abnormal (high) T-tau (A+ T– (N)+), *n* = 17 (13%); and both abnormal P-tau and T-tau (A+ T+ (N)+), *n* = 42 (33%).

**Table 1:** Socio-demographic and clinical characteristics by AT(N) biomarker profiles (*n* = 129)

	A+ T– (N)– (*n* = 58, 45%)	A+ T+ (N)– (*n* = 12, 9%)	A+ T– (N)+ (*n* = 17, 13%)	A+ T+ (N)+ *(n* = 42, 33%)	*p* value
Variable	*n*/%	*n*/%	*n*/%	*n*/%	
Female sex	34/59%	10/83%	14/82%	30/71%	0.139
Carrier of the *APOE* ε4 allele	41/71%	8/67%	10/59%	34/81%	0.340
Type of ChEI agent					0.445
Donepezil (*n* = 71)	37/64%	8/67%	7/41%	19/45%	
Rivastigmine (*n* = 24)	9/15%	1/8%	4/24%	10/24%	
Galantamine (*n* = 34)	12/21%	3/25%	6/35%	13/31%	
Variable	Mean ± standard deviation				*p* value
Estimated age at onset, years	72.9 ± 7.2	74.5 ± 4.8	77.1 ± 5.7	70.3 ± 6.2	0.003
Estimated duration of AD at baseline, years	3.2 ± 2.4	2.3 ± 1.4	2.2 ± 1.3	3.0 ± 1.9	0.228
Age at baseline, years	76.1 ± 6.2	76.8 ± 4.6	79.2 ± 6.2	73.3 ± 6.0	0.005
Education, years	10.2 ± 3.1	9.4 ± 1.9	9.1 ± 2.0	9.0 ± 2.0	0.116
MMSE score at baseline	21.7 ± 3.8	19.5 ± 3.7	20.6 ± 4.6	20.2 ± 4.0	0.181
ADAS-cog score (0–70) at baseline	20.8 ± 9.1	21.8 ± 9.9	20.6 ± 10.2	23.2 ± 9.3	0.633
IADL score at baseline	17.2 ± 5.7	14.6 ± 5.9	15.9 ± 5.7	15.9 ± 4.9	0.403
PSMS score at baseline	7.9 ± 2.9	7.2 ± 1.9	8.3 ± 3.1	7.4 ± 2.2	0.568
Number of concomitant medications at baseline	3.5 ± 2.8	3.3 ± 3.6	3.5 ± 2.8	2.6 ± 2.1	0.335
Aβ_42,_ ng/ml	122 ± 22	116 ± 12	118 ± 19	115 ± 14	0.274
T-tau, ng/ml	72 ± 15	82 ± 11	122 ± 19	155 ± 46	< 0.001
P-tau, ng/ml	30 ± 13	61 ± 8	40 ± 9	79 ± 24	< 0.001

Abbreviations: *A+* abnormal CSF Aβ_42_; *Aβ*_*42*_ amyloid-β_1–42_; *AD* Alzheimer’s disease; *ADAS-cog* Alzheimer’s Disease Assessment Scale-cognitive subscale; *APOE*, apolipoprotein E; *ChEI* cholinesterase inhibitor; *IADL* Instrumental Activities of Daily Living scale; *MMSE* Mini-Mental State Examination; *(N)–* normal CSF T-tau; *(N)+* abnormal CSF T-tau; *PSMS* Physical Self-Maintenance Scale; *P-tau* phosphorylated tau; *T–* normal CSF P-tau; *T+* abnormal CSF P-tau; *T-tau* total tau

The individuals with A+ T+ (N)+ were younger at the estimated onset of AD (F_3,125_ = 4.78, *p =* 0.003) and at the start of ChEI treatment (baseline) (F_3,125_ = 4.46, *p =* 0.005) than those with A+ T– (N)+. As expected, the levels of P-tau (F_3,125_ = 73.68, *p <* 0.001) and T-tau (F_3,125_ = 68.57, *p <* 0.001), but not Aβ_42_, differed between the AT(N) biomarker profiles. Post hoc tests (Bonferroni) showed significant differences for all pairwise comparisons of P-tau with the exception of the combination of A+ T– (N)– and A+ T– (N)+, and for all pairwise comparisons of T-tau except for the combination of A+ T– (N)– and A+ T+ (N)–. However, no baseline differences in estimated duration of AD, education level, cognitive and functional capacities, and number of concomitant medications were found between the AT(N) profiles (Table 1).

**Fig. 1:**
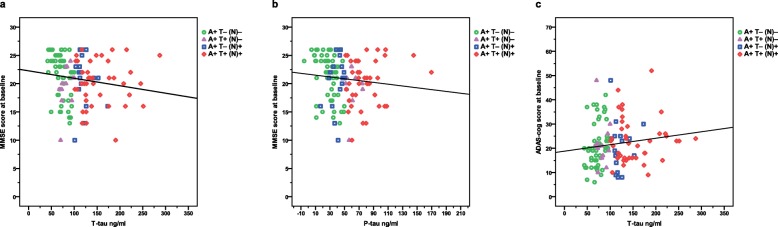
Cognitive status at baseline and CSF T-tau or P-tau by AT(N) biomarker profiles. **a** More impaired MMSE score at the start of ChEI therapy (time of AD diagnosis) showed a linear association with higher T-tau (*r*_*s*_ = − 0.222, *p =* 0.012). **b** A negative correlation between the SATS patients’ MMSE score and P-tau (*r*_*s*_ = − 0.204, *p* = 0.020) was also observed. **c** Worse ADAS-cog score at baseline demonstrated a linear relationship with higher T-tau (*r*_*s*_ = 0.194, *p =* 0.030). Abbreviations: A+, abnormal CSF Aβ_42_; AD, Alzheimer’s disease; ADAS-cog, Alzheimer’s Disease Assessment Scale–cognitive subscale; ChEI, cholinesterase inhibitor; CSF, cerebro-spinal fluid; MMSE, Mini-Mental State Examination; (N)–, normal CSF T-tau; (N)+, abnormal CSF T-tau; P-tau, phosphorylated tau; SATS, Swedish Alzheimer Treatment Study; T–, normal CSF P-tau; T+, abnormal CSF P-tau; T-tau, total tau

Using the continuous CSF biomarker values, linear relationships were observed between more impaired cognition at the initiation of ChEIs measured by MMSE score and higher P-tau (*r*_*s*_ = − 0.204, *p* = 0.020) or T-tau (*r*_*s*_ = − 0.222, *p =* 0.012), and between ADAS-cog score and T-tau (*r*_*s*_ = 0.194, *p =* 0.030) (Fig. 1 a–c). No significant correlations were detected between ADL performance and P-tau or T-tau, or between Aβ_42_ and any of the baseline measures.

### Short-term response to ChEI therapy and longitudinal outcomes

The proportion of SATS participants who showed improvement/no change (≥ 0 point change) after 6 months of ChEI treatment did not differ between the four AT(N) biomarker profiles in any of the scales (Table 2).

**Table 2:** Changes in cognitive and functional abilities by AT(N) biomarker profiles during 3 years of ChEI therapy

Variable	A+ T– (N)–	A+ T+ (N)–	A+ T– (N)+	A+ T+ (N)+	*p* value
*Response to ChEIs*					
MMSE score, improved/unchanged patients after 6 months (%)	62	91	53	54	0.141
ADAS-cog score (0–70), improved/ unchanged patients after 6 months (%)	62	18	56	55	0.077
IADL score, improved/unchanged patients after 6 months (%)	53	36	25	41	0.218
PSMS score, improved/unchanged patients after 6 months (%)	55	91	63	68	0.141
*Rates of progression*	Mean (95% confidence interval)				*p* value
MMSE score, decline/year	−1.7 (−3.1, −0.2)	−1.4 (−3.0, 0.3)	−1.1 (−2.0, −0.1)	−2.9 (−4.1, − 1.8)	0.361
ADAS-cog score (0–70), decline/year	−1.7 (−3.3, −0.2)	−2.7 (−4.5, −1.0)	−2.8 (−5.1, −0.4)	−3.9 (−5.6, −2.1)	0.286
IADL score, decline/year	−2.6 (−3.3, −1.9)	−3.1 (−4.8, − 1.5)	−2.8 (− 3.9, − 1.8)	−4.2 (− 5.7, − 2.7)	0.148
PSMS score, decline/year	−1.7 (−2.4, −0.9)	−1.4 (−2.6, − 0.1)	− 1.2 (− 1.9, − 0.5)	−1.4 (− 2.2, − 0.7)	0.891
Length in the SATS, months	22.9 (19.6, 26.3)	27.5 (20.3, 34.7)	31.1 (27.1, 35.0)	23.8 (19.9, 27.7)	0.081

For clarity, clinical improvements for all scales have been tabulated as positive changes from the start of ChEI therapy (approximately time of AD diagnosis)

Abbreviations: *A+* abnormal CSF Aβ_42_; *AD* Alzheimer’s disease; *ADAS-cog* Alzheimer’s Disease Assessment Scale-cognitive subscale; *ChEI* cholinesterase inhibitor; *IADL* Instrumental Activities of Daily Living scale; *MMSE* Mini-Mental State Examination; *(N)–* normal CSF T-tau; *(N)+* abnormal CSF T-tau; *PSMS* Physical Self-Maintenance Scale; *SATS* Swedish Alzheimer Treatment Study; *T–* normal CSF P-tau; *T+* abnormal CSF P-tau

By using the continuous CSF biomarker values and 6-month cognitive or functional changes in scores from baseline, no linear associations between any biomarker and the response to ChEIs were found. Patients with the lowest quartile and quintile of Aβ_42_ (≤ 104 and ≤ 106 ng/ml), and the highest quartile and quintile of P-tau (≥ 65 and ≥ 70 ng/ml) and T-tau (≥ 126 and ≥ 129 ng/ml), respectively, were also examined; their response to ChEIs did not differ from the groups with less pronounced pathological biomarkers.

**Fig. 2:**
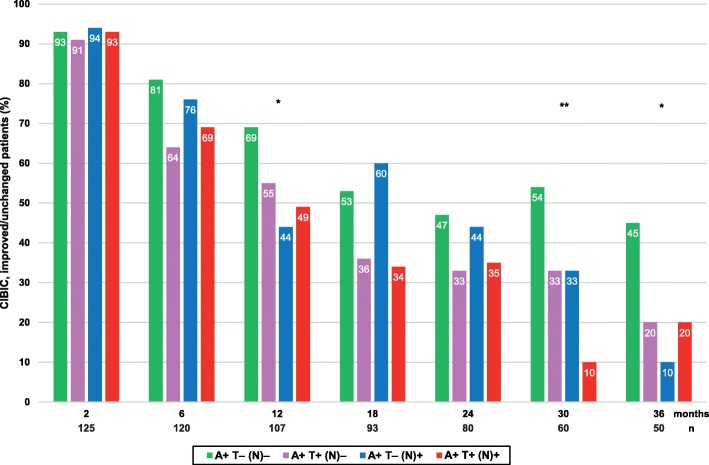
Global response over 3 years of ChEI treatment. The proportion of improved/unchanged participants in global performance (CIBIC score, 1–4) from the initiation of ChEIs over 3 years according to AT(N) biomarker profile. A higher frequency of improved/unchanged patients was exhibited in A+ T– (N)– after 12 months (*p* = 0.034), 30 months (*p* = 0.005) and 36 months (*p* = 0.029) of therapy. No significant difference in AT(N) profiles was found between the improved vs. the unchanged individuals at any assessment. Abbreviations: A+, abnormal CSF Aβ_42_; ChEI, cholinesterase inhibitor; CIBIC, Clinician Interview-Based Impression of Change; CSF, cerebro-spinal fluid; (N)–, normal CSF T-tau; (N)+, abnormal CSF T-tau; T–, normal CSF P-tau; T+, abnormal CSF P-tau

A higher percentage of globally improved/unchanged individuals (CIBIC score: 1–4) was exhibited in A+ T– (N)– compared with the other patients after 12 months (*p* = 0.034), 30 months (*p* = 0.005) and 36 months (*p* = 0.029) of ChEI therapy, but not at the other evaluations (Fig. 2). The annual decline in cognition or ADL did not differ between the AT(N) biomarker profiles in any of the scales (Table 2). Separate analyses of the rates of change/year in the two most complex IADL items (‘responsibility for own medications’ and ‘ability to handle finances’) revealed no significant differences between the AT(N) groups. Moreover, the quartile or quintile of participants with the most abnormal values of the CSF biomarkers (Aβ_42_, P-tau or T-tau) showed similar longer-term cognitive and functional impairment as those in the other quartiles or quintiles. The mean percentage of the maximum recommended ChEI dose during the SATS, i.e., 10 mg for donepezil, 12 mg for rivastigmine, and 24 mg for galantamine did not differ between the four AT(N) profiles (F_3,125_ = 0.41, *p =* 0.747).

Using the continuous biomarker values, linear relationships were observed between greater annual deterioration in MMSE score and higher T-tau (*r*_*s*_ = − 0.183, *p* = 0.040), and between faster progression rate in ADAS-cog score and higher P-tau (*r*_*s*_ = − 0.242, *p* = 0.007)**.** A linear association was also found between more rapid worsening in IADL score (but not basic ADL) and higher T-tau (*r*_*s*_ = − 0.184, *p* = 0.040). No significant correlations were demonstrated between cognitive or functional decline per year and Aβ_42_.

After 3 years, 50 patients (39%) completed the study; A+ T– (N)–, *n* = 20 (34%); A+ T+ (N)–, *n* = 5 (42%); A+ T– (N)+, *n* = 10 (59%); and A+ T+ (N)+, *n* = 15 (36%), (χ^2^(3) = 3.54; *p =* 0.316). The reasons for dropout were initiation of concomitant memantine therapy (*n* = 18, 14%), withdrawal of informed consent (*n* = 11, 8%), side effects (*n* = 10, 8%), admission to nursing homes (*n* = 7, 5%), switching to another ChEI agent (*n* = 6, 5%), poor effect/deterioration (*n* = 6, 5%), death (*n* = 4, 3%), compliance problems (*n* = 4, 3%), switching to another study (*n* = 3, 2%), somatic disease assumed to be unrelated to AD (*n* = 1, 1%), and other reasons (*n* = 9, 7%). The mean time of participation in the SATS did not differ between the AT(N) biomarker profiles (F_3,125_ = 2.29, *p =* 0.081) (Table 2). The drop-outs had lower cognitive status at the start of ChEI treatment compared with the completers (mean ± standard deviation (SD), MMSE: 20.1 ± 4.2 vs. 22.0 ± 3.4 points; t_127_ = 2.75; *p* = 0.007, and ADAS-cog: 23.6 ± 9.9 vs. 18.4 ± 7.4 points; t_124_ = − 3.37; *p* = 0.001). The characteristics of sex, *APOE* genotype, age at onset and years of education, as well as age, ADL capacity, number of concomitant medications and CSF biomarker levels at baseline were similar between the two groups.

### Outcomes according to normal P-tau (T–) vs. abnormal P-tau (T+)

The individuals with T– (A+ T– (N)– or A+ T– (N)+) were older at the estimated onset of AD (mean ± SD, 73.9 ± 7.1 vs. 71.2 ± 6.1 years; t_127_ = 2.22, *p* = 0.028) and at the initiation of ChEI therapy (76.8 ± 6.3 vs. 74.1 ± 5.8 years; t_127_ = 2.53, *p* = 0.013) than those with T+ (A+ T+ (N)– or A+ T+ (N)+). A trend towards higher Aβ_42_ among the T– compared with the T+ group was shown (121 ± 21 vs. 116 ± 14 ng/ml; t_127_ = 1.93, *p* = 0.055). As expected, the levels of tau were lower in patients with T– than in those with T+, (P-tau: 32 ± 13 vs. 75 ± 23 ng/ml; t_127_ = − 12.52, *p* < 0.001; and T-tau: 83 ± 26 vs. 139 ± 51 ng/ml; t_127_ = − 7.31, *p* < 0.001). No significant differences in sex, *APOE* genotype, duration of AD, years of education, cognitive and functional status at baseline, and use of medications were exhibited between the two groups. The effects of T– vs. T+ on short-term response to ChEIs and annual rates of decline were also analysed, but no differences between the groups on any scales were detected.

### Outcomes according to *APOE* genotype

**Fig. 3:**
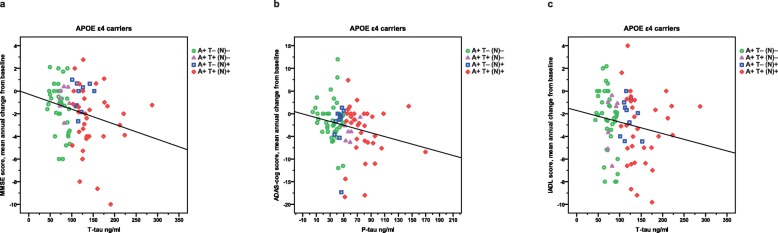
Cognitive and functional decline per year and CSF T-tau or P-tau in *APOE* ε4 carriers by AT(N) biomarker profiles. **a** More pronounced annual deterioration in MMSE score showed a linear association with higher T-tau at the start of ChEI therapy (time of AD diagnosis) (*r*_*s*_ = − 0.257, *p* = 0.014). **b** Decline per year in ADAS-cog score demonstrated a negative correlation with P-tau at baseline (*r*_*s*_ = − 0.242, *p* = 0.022). **c** A linear relationship between faster progression rate in IADL score/year and higher T-tau was also found (*r*_*s*_ = − 0.232, *p* = 0.028). These correlations were not significant among the *APOE* non-ε4 carriers. Abbreviations: A+, abnormal CSF Aβ_42_; AD, Alzheimer’s disease; ADAS-cog, Alzheimer’s Disease Assessment Scale–cognitive subscale; *APOE*, apolipoprotein E; ChEI, cholinesterase inhibitor; CSF, cerebro-spinal fluid; IADL, Instrumental Activities of Daily Living Scale; MMSE, Mini-Mental State Examination; (N)–, normal CSF T-tau; (N)+, abnormal CSF T-tau; P-tau, phosphorylated tau; T–, normal CSF P-tau; T+, abnormal CSF P-tau; T-tau, total tau

The level of Aβ_42_ was lower among the *APOE* ε4 carriers than the non-ε4 carriers (mean ± SD, 116 ± 16 vs. 125 ± 22 ng/ml; t_127_ = 2.52; *p* = 0.013). No differences in sex, age at onset or at baseline, years of education, cognitive and functional status at baseline, use of medications and tau were observed between the two groups. Among the *APOE* ε4 carriers, a linear relationship was found between lower MMSE score (but not ADAS-cog) at the start of ChEIs and higher P-tau (*r*_*s*_ = − 0.216, *p* = 0.038) and a trend towards T-tau (*r*_*s*_ = − 0.202, *p* = 0.053). No correlations between the CSF biomarkers and functional performance at baseline were demonstrated. Linear associations were also shown among the ε4 carriers between faster worsening in MMSE score and higher T-tau (*r*_*s*_ = − 0.257, *p* = 0.014), more rapid deterioration in ADAS-cog score and higher P-tau (*r*_*s*_ = − 0.242, *p* = 0.022), progression rate in IADL score (but not basic ADL) and higher T-tau (*r*_*s*_ = − 0.232, *p* = 0.028) (Fig. 3 a–c), and a trend towards P-tau (*r*_*s*_ = − 0.200, *p* = 0.059). The aforementioned correlations were not significant among the *APOE* non-ε4 carriers.

The interaction effects of normal/abnormal levels of tau with presence/absence of the *APOE* ε4 allele on response to ChEIs, annual rates of decline and 3-year mean change in scores were also analysed. No significant differences between the four groups in any scales were detected.

## Discussion

In this observational, long-term study, we reported that the participants with A+ T+ (N)+ (pathologic tau and neurodegeneration) were younger at the estimated onset of AD and at the initiation of ChEI treatment than those with A+ T– (N)+ (normal tau but with neurodegeneration). The estimated duration of AD, cognitive and functional ability, number of medications, and the level of Aβ_42_ at baseline did not differ between the AT(N) biomarker profiles. However, linear relationships were exhibited between more impaired cognition at baseline and higher P-tau or T-tau. Among *APOE* ε4 carriers, greater annual worsening in MMSE or IADL scores was associated with higher T-tau. A significant correlation was also found between more rapid ADAS-cog decline per year and higher P-tau. These linear associations were not significant among the non-ε4 carriers.

Previous studies of levels of CSF biomarkers have suggested that Aβ_42_ reflects the amount of amyloid plaques in the brain “A”; thus, the aggregation of beta-amyloid into plaques results in decreased availability of beta-amyloid in the CSF. Furthermore, P-tau reflects the formation of neurofibrillary tangles “T”, while T-tau is assumed to mirror the intensity of neuronal degeneration and brain damage, which is not AD specific “(N)” [38]. Consequently, relationships between the more advanced AD severity and the lower levels of Aβ_42_ as well as higher levels of P-tau and T-tau are expected. Recently, a research framework AT(N) was created to facilitate a biomarker-based definition of the three aforementioned pathological processes (A, T, (N)) using a cut-off for each pathology (normal/abnormal) in living persons [8].

In the present study, almost half of the clinically diagnosed AD patients had normal tau and no sign of neurodegeneration measured by CSF T-tau; noticeably, the cognitive and functional changes in scores over time did not differ from those with pathologic tau and neurodegeneration. Prior studies demonstrated that not all persons with AD have a clear abnormal pattern of all three CSF biomarkers [41]. A common framework, such as the AT(N) for defining and staging the disease by imaging or CSF biomarkers, might facilitate standardized reporting of research findings across the field [8]. One study published before the AT(N) described five subgroups with different biomarker profiles; one of the AD groups was characterized by individuals with high incidence of Lewy bodies, abnormal CSF Aβ_42_, normal T-tau (P-tau was not addressed) and late onset and older age [41]. Similarly, the participants with pathologic tau and neurodegeneration in our study were younger at onset of AD and at baseline, indicating more hereditary and aggressive subtypes of the disease. Although the AT(N) classification is not intended for routine clinical care, the diversity of possible CSF biomarker profiles that accompany AD might be unclear for clinicians, hence complicating the interpretation of biomarker results and making a diagnosis in daily clinical practice.

Some longitudinal AD studies have observed that the T-tau level was increased at follow-up visits of the patients [18, 19], while others noted stability of tau over time, despite changes in the individual’s cognitive and global status [14, 17]. A recent study that included a small-sample mild AD cohort from the ADNI database showed a decreased annual change of the more specific AD marker P-tau, but not of T-tau. A slowing of the neurodegenerative process might reflect neuronal death, i.e., the smaller number of neurons that remain in the brain [20]. Taken together, the association between the levels of CSF biomarkers and disease severity or rate of progression is inconsistent and not well understood. An explanation of the various findings might be that the CSF biomarker levels, especially of Aβ_42_, seem to be substantially altered very early in the disease process, many years before the symptoms occur, which might imply weak correlations between the stage of AD and the biomarker values [14, 17, 42].

In the current study, weak but significant linear correlations (*r*_*s*_ = − 0.22 to − 0.19) were exhibited between lower cognitive (but not functional) capacity at baseline and higher CSF P-tau or T-tau. Other studies of AD have also reported similar cross-sectional relationships between the participants’ cognitive test results and level of tau [42, 43]; however, an earlier small-sample study demonstrated an even stronger correlation between lower MMSE score and higher T-tau (*r* = − 0.66) [44]. Nevertheless, not all previous studies showed associations between baseline cognitive performance and CSF tau [11, 17, 45]. The individuals in the SATS and in the aforementioned studies [11, 17, 42, 43] were in the mild-to-moderate AD stages (mean MMSE score: 20–23); therefore, changes in the biomarkers early in the disease process might not be the only explanation for these various observations. CSF biomarkers could be valuable in the preclinical phase, but the inconsistency among the different studies may reflect the variation of biomarker levels at these stages of AD.

Genetic and demographic factors might also affect the outcome of biomarkers. The *APOE* ε4 carriers in this study had more pronounced pathological levels of CSF Aβ_42_ (but not tau), which may suggest more hereditary and advanced forms of the disease. A recent study described a greater tau aggregation in the temporal and parietal lobes among ε4 carriers compared with non-ε4 carriers [46]. Among the *APOE* ε4 carriers (but not among the non-ε4 carriers) in the SATS, we found relationships between higher tau and faster cognitive or IADL deterioration. The ability of tau to stabilize microtubules has been reported to be impaired by the presence of the *APOE* ε4 allele, leading to a shorter function and survival of the neurons [47]. However, the associations between *APOE* genotype, CSF biomarkers and AD prognosis are not clear. A more rapid cognitive progression rate and a higher increase of tau over time among ε4 carriers in comparison with non-ε4 carriers have been demonstrated [48]. Another AD study observed no correlations between cognitive or global measures, *APOE* genotype and T-tau [49]. The patients in both these studies [48, 49] had similar cognitive status at baseline (mean MMSE score 15–18), i.e., lower than in our SATS cohort (mean MMSE score 21); hence, cognitive performance cannot explain the inconsistent findings. In addition, the education level in the US cohort [49] was very high (mean 15 years vs. SATS 10 years), indicating that those individuals might have a higher cognitive reserve capacity and thus a more advanced disease in relation to their outcomes on cognitive tests, which might impair the ability to detect potential statistical relationships. Conflicting results in the same AD study, such as decreased CSF tau levels in participants with two *APOE* ε4 alleles and increased tau levels in those with one ε4 allele, compared with non-ε4 carriers, have also been shown [50]. Based on these mixed observations, it is not possible to conclude whether the levels of CSF tau correlate with *APOE* pathogenesis and cognitive progression.

In this study, a linear correlation was also exhibited among the *APOE* ε4 carriers between faster IADL decline per year and higher T-tau. Very few studies have investigated the potential associations between CSF biomarkers, *APOE* genotype and ADL, despite the fact that worsening in daily functioning is commonly the most troubling aspect of AD for patients and family members, and disabilities in IADL are considered to be a predominant critical factor behind community-based services (e.g., home help and nursing home placement) and thus increasing societal costs [30, 31]. Surprisingly, two earlier studies using data from the ADNI came to inconsistent conclusions regarding the relationship between CSF biomarkers and ADL in mild AD. Okonkwo et al. [15] found that no biomarker was predictive of functional deterioration (the impact of *APOE* genotype was not addressed), while Marshall et al. [16] reported associations between impairment in IADL over time and lower Aβ_42_ and higher T-tau (these were independent of *APOE* status). Both ADNI studies demonstrated a similar frequency of *APOE* ε4 carriers (66–69%); therefore, the stage of AD and the selection of individuals cannot explain the different outcomes. The SATS included a slightly higher percentage of ε4 carriers (72%), which might entail more hereditary influence and decreasing functional capacity. No correlation between rate of progression in IADL score and CSF Aβ_42_ was detected in the present study; however, the SATS participants were in the mild-to-moderate stage of AD and all patients showed abnormal Aβ_42_ at baseline. In preclinical AD using the AT(N) framework, the onset of driving problems was mainly associated with presence of both amyloid and tau pathology [51]. A review observed that IADL deficiencies occurred early during the stage of mild cognitive impairment [52] that underlines the need for functional assessments during the milder phases of cognitive decline. Our results may indicate that evidence of pathologic tau and/or neuronal injury significantly contributes to the worsening of long-term functional performance in AD, particularly in *APOE* ε4 carriers.

The response to ChEI therapy after 6 months did not differ between the AT(N) biomarker profiles on any of the scales, indicating that in patients with both dementia and accumulation of amyloid in the brain, the absence or presence of pathologic tau or neuronal injury could not predict treatment response. A more positive cognitive response to ChEIs in AD has been described in participants with lower cognitive ability at the start of therapy [22, 23, 32], which suggests more advanced neurodegeneration in the brain. Very few studies have evaluated the associations between response to ChEIs and CSF biomarkers and the findings were inconsistent. An earlier AD study from our Memory Clinic using cluster analysis reported worse cognitive short-term response to ChEI treatment in the cluster that included patients with very high levels of P-tau and T-tau, i.e., a more aggressive disease [24]. Another study, also from our group, used binary logistic regression and found that the levels of CSF biomarkers did not predict response to ChEI therapy [32]. ChEIs might be more effective for individuals in a stage of AD with greater cognitive disabilities and cholinergic dysfunction, but less beneficial for those with a more intense disease and faster neuronal loss [24, 53]. Different statistical methods and sensitivity of the scales used could also have an impact on the results. Socio-economic and clinical predictors can also affect short-term treatment response to ChEIs and longer-term prognosis of AD [23]. However, the relationships between biomarkers and clinical treatment response seem weak. Phase 3 trials of passive immunization with anti-beta-amyloid antibodies have demonstrated reduced beta-amyloid in the brain, but no significant clinical effects [25, 26]. A stronger correlation has been shown between cognitive deficits and the extent of neurofibrillary tangles than between cognition and density of amyloid plaques [6]. Therefore, drug agents directed towards tau might be a better therapeutic target in AD. Clinical trials of immunization with anti-tau antibodies are now ongoing and the results remain to be seen [27].

The strengths of the observational, prospective SATS are the well-structured, 6-month assessments of different aspects of AD progression over 3 years after the initiation of ChEI treatment; however, CSF biomarkers were only measured at baseline. Everyday outpatients with concomitant disorders and medications were included, and the participants were diagnosed and followed up by specialists in dementia disorders at the Memory Clinic. The CSF biomarkers were analysed after the clinical follow-up was completed, yet all individuals had abnormal Aβ_42_ assuming accuracy of the AD diagnosis. Like other longitudinal naturalistic studies of AD, the limitations are that the SATS was not placebo controlled because of ethical concerns, or randomized with respect to ChEI agent, and that drop-out occurred over the study period. The 3-year completion rate of 39% was high compared with other AD extension or naturalistic studies (20–39%) [54]. Moreover, all patients contributed with data during their time of participation. The drop-out cohort exhibited lower cognitive performance at baseline than the completers, but the other characteristics including the levels of CSF biomarkers were similar between the groups. Functional capacities were assessed using informant-based scales. These might have limitations if perceptions and opinions are introduced into the informant’s report on the care recipient’s ADL status; however, experienced dementia nurses interviewed the caregivers in the SATS. In addition, informant-based scales are widely used in dementia drug trials and usually have good reliability and validity [55].

Future studies of the associations of CSF biomarkers with clinically relevant measures (cognition, global status, ADL), as well as their possible associations with *APOE* genotype, are needed to increase the knowledge of these still unclear relationships. The AT(N) framework needs to be thoroughly examined by both randomized clinical trials and observational studies and possibly modified before being adopted into routine clinical settings. Patients with dissimilar biomarker profiles, and probably various causes and mechanisms of neurodegeneration, might respond differently to therapeutic drugs. Despite reduced amyloid burden in the brain after immunization therapy, no significant clinical improvement or stabilization in individuals with AD has been detected so far. Continued investigations of the AD pathologies and their suggested downstream effects in subgroups are essential for the development of new treatments.

## Conclusions

In this clinical practice-based, long-term study, almost half of the participants with AD did not exhibit pathologic tau or neuronal injury. The variety of CSF biomarker patterns that can accompany the disease may contribute to the challenge of interpreting biomarkers and improving diagnostic certainty in clinical routine. The individuals with pathologic tau and neurodegeneration were younger, indicating a more aggressive disease. No associations between the levels of any biomarker and the short-term response to ChEI therapy in cognitive, global or ADL performance were found, showing a low correlation between AD pathologies and clinical treatment response. A more pronounced global, but not cognitive, rate of progression was demonstrated in patients with pathologic tau and/or neuronal injury, which might indicate worse prognosis in this group. However, among the *APOE* ε4 carriers exclusively, greater annual MMSE, ADAS-cog or IADL decline were associated with higher P-tau or T-tau, which suggests a relationship between these risk factors leading to a subgroup of patients with more rapid cognitive and/or functional deterioration. The IADL is an important measure in AD, and these observations stress the importance for the clinician to evaluate IADL status to predict the individual’s ability to manage independently over time. Our results indicate that the AT(N) biomarker profiles have limited utility in measuring response in clinical trials and progression rates over longer periods in mild-to-moderate AD. These findings might be useful when considering new diagnostic criteria and when interpreting outcomes from future clinical trials of potentially disease-modifying AD therapies.

## Data Availability

Currently, we are unable to share the SATS data because data collection, such as dates of death and the patients’ previous diagnoses, is presently taking place and the data analysis process is still ongoing.

## Abbreviations

(N)+: Abnormal CSF T-tau
(N)–: Normal CSF T-tau
A+: Abnormal CSF Aβ_42_
ACh: Acetylcholine
AD: Alzheimer’s disease
ADAS-cog: Alzheimer’s Disease Assessment Scale–cognitive subscale
ADL: Activities of Daily Living
ANOVA: One-way analysis of variance
*APOE*: Apolipoprotein E
Aβ_42_: Amyloid-β_1–42_
ChEI: Cholinesterase inhibitors
CI: Confidence interval
CIBIC: Clinician Interview-Based Impression of Change
CSF: Cerebro-spinal fluid
IADL: Instrumental Activities of Daily Living Scale
LP: Lumbar puncture
MMSE: Mini-Mental State Examination
PSMS: Physical Self-Maintenance Scale
P-tau: Phosphorylated tau
SATS: Swedish Alzheimer Treatment Study
SD: Standard deviation
T+: Abnormal CSF P-tau
T–: Normal CSF P-tau
T-tau: Total tau
